# First report of southern root-knot nematode, *Meloidogyne incognita*, infecting *Brassica nigra* in Peru

**DOI:** 10.21307/jofnem-2020-114

**Published:** 2020-10-21

**Authors:** Jorge Airton Gómez-Chatata, Juan José Tamo-Zegarra, Teodocia Gloria Casa-Ruiz, Cristiano Bellé

**Affiliations:** 1 Universidad Nacional de San Agustin de Arequipa, Arequipa, Peru; 2 Universidade Federal de Santa Maria, Rio Grande do Sul, Santa Maria, Brazil; 3 Instituto Phytus, Estação experimental de Itaara, Itaara, Rio Grande do Sul, Brazil

**Keywords:** Detection, Diagnosis, Identification

## Abstract

*Brassica nigra* plants showing symptoms caused by root-knot nematodes were detected in the municipality of La Joya, Arequipa Province, Peru. Based on morphology, esterase phenotypes, and species-specific characterized amplified region (SCAR) sequence, the causal agent was identified as *Meloidogyne incognita*. Pathogenicity was confirmed by a modified version of Koch’s postulates. To our knowledge, this is the first report of *M. incognita* infecting *Brassica nigra* in Peru.

*Brassica nigra* (L.) W.D.J. Koch (black mustard) is important as a crop plant; it may have contributed to the evolution of several species in the genus *Brassica* (El-Esawi, 2017). It is widely cultivated, mostly continents as Australasia and the Americas (Rakow, 2004). Black mustard was important oil seed crop, and has potential for use as green manure crops.

Plants attacked by pests, diseases, and plant-parasitic nematodes can impair production both qualitatively and quantitatively (Sikora et al., 2018). Among the plant-parasitic nematodes, the most important genus is *Meloidogyne* (Göldi, 1887), which causes damage in the form of root galls and may reduce in the number of roots, and predispose the plant to fungal and bacterial diseases causing losses in crop yields (Karssen, 2002; Sikora et al., 2018).

In February 2020, many nematode galls (Fig. 1A, B) were observed on the roots of black mustard plants, and samples were taken from areas (16°27′43.5″S; 71°49′19.6″W), in La Joya, Arequipa Province, Peru. In order to identify the plant-parasitic nematode species, a combination of morphological, biochemical, and molecular analyses were performed.

**Figure 1:**
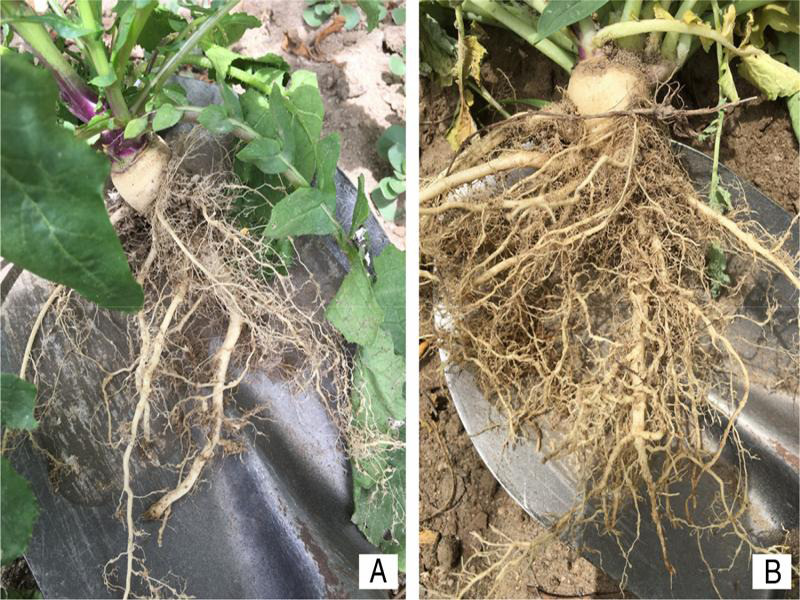
A and B: Roots of *Brassica nigra* (L.) W.D.J. Koch showing galls induced by *Meloidogyne incognita* (Kofoid and White, 1919; Chitwood, 1949).

This population of root-knot nematode was identified to species with esterase phenotypes (*n* = 36 females) (Carneiro and Almeida, 2001); morphology, and morphometrics of second-stage juveniles (J2) (*n* = 30), females (*n* = 10), and perineal patterns (*n* = 15); species molecular identification was confirmed by PCR species-specific characterized amplified region (SCAR) sequence for confirmation, using a primer set composed of inc-K14-F (5′-GGGATGTGTAAATGCTCCTG-3′) and inc-K14-R (5′-CCCGCTACACCCTCAACTTC-3′) (Randig et al., 2002).

The nematode population density was 1,405 J2/g of root. Morphometrics (means followed by ± standard deviation with the minimum and maximum values in parentheses) of J2s: length (*L*) = 360.7 ± 21.3 (310-476) μm, *a* = 22.1 ± 4.3 (20.3-25.5), *c* = 8.4 ± 0.5 (4.9-9.5), stylet length = 11.3 ± 0.5 (9.1-12.2) μm, dorsal esophageal gland orifice to base of stylet (DGO) = 2.5 ± 0.4 (1.7-2.6) μm, tail length = 39.5 ± 1.0 (39.0-48.5) μm and hyaline tail terminus = 10.5 ± 0.5 (9.5-11.2) μm. Morphometrics of females: *L* = 655.5 ± 30.0 (550.4.5-700.5) μm, stylet length = 14.5 ± 0.5 (12.1-15.4) μm, and DGO = 3.5 ± 0.2 (2.8-4.0) μm. The perineal pattern of the female included a high and square dorsal arch with wavy striae bending toward area the lateral lines and the absence of distinct lateral line incisures. The overall morphology and morphometrics of this population appears similar to that of *Meloidoyne incognita* (Kofoid and White, 1919; Chitwood, 1949; Hunt and Handoo, 2009).

The polymorphisms of the esterase bands by electrophoresis revealed the phenotype I2 (Rm = 1.05 and 1.10) typical of *M. incognita* (Carneiro et al., 1996). The PCR amplification using SCAR sequence produced a specific fragment of expected size (∼399 bp) for *M. incognita* (Randig et al., 2002).

In greenhouse tests, *Brassica nigra* plantlets were maintained in pots with 2 liters sterilized soil. In total, six replicates were inoculated with 3,000 eggs and J2s from the original population of *M. incognita*, in addition to a non-inoculated control. Eggs and J2 were extracted from infected roots using the 0.5% NaOCl (Hussey and Barker, 1973) method as modified by Boneti and Ferraz (1981). Plants were well maintained under greenhouse conditions at 25 ± 3°C. After 60 days, the inoculated plants exhibited galled root systems similar to plants observed in the field, with a nematode reproduction factor (final population/initial population) of 23.5. The non-inoculated plants did not exhibit any galls. The morphological and molecular characterization of this root-knot nematode were identical to those of *M. incognita*.

To our knowledge, this is the first report of *M. incognita* parasitizing *Brassica nigra* in Peru. This finding may be important to Peruvian agriculture, since this nematode may damage black mustard plants becoming an additional problem for this crop. Additional surveys of black mustard may warrant the development of adequate control strategies for root-knot nematode if this nematode is widespread.
